# Effects of liarozole fumarate (R85246) in combination with tamoxifen on N-methyl-N-nitrosourea (MNU)-induced mammary carcinoma and uterus in the rat model

**DOI:** 10.1186/1471-2407-7-26

**Published:** 2007-01-31

**Authors:** Paul E Goss, Kathrin Strasser-Weippl, Shangle Qi, Haiqing Hu

**Affiliations:** 1 Breast Cancer Research, Massachusetts General Hospital Cancer Center, Breast Cancer Disease Program, Dana Farber/Harvard Cancer Center, Harvard Medical School, Boston, Massachusetts, USA; 2 1^st ^Medical Department – Center for Hematology and Medical Oncology, Wilhelminen Hospital, Vienna, Austria

## Abstract

**Background:**

Liarozole fumarate (liarozole – R85246) is a novel compound with characteristics of both aromatase inhibitor (AI) and a retinoic acid metabolism blocking agent (RAMBA). Our objective was to determine the effects of liarozole alone or in combination with tamoxifen on the N-methyl-N-nitrosourea (MNU)-induced rat mammary carcinoma model, as well as on the uterus in ovariectomized immature rats.

**Methods:**

(1) Tumor burden experiments: Animals bearing one or more tumors greater than 10 mm in diameter were treated for 56 consecutive days with 20 mg/kg or 80 mg/kg of liarozole by oral gavage, tamoxifen 100 μg/kg by subcutaneous injection, or a combination of liarozole and tamoxifen. At the end of the treatment period, total cumulative tumor volume as well as retinoic acid levels were measured. (2) Uterotrophic assay and proliferation experiments: 21-day-old ovariectomized (OVX) Sprague-Dawley rats were treated with 20 mg/kg or 80 mg/kg of liarozole by oral gavage, tamoxifen 1 mg/kg by subcutaneous injection, and combination of both for 4 consecutive days. At the end of the treatment period, uterine weight, epithelial lining cell height and indices of proliferation cell nuclear antigen (PCNA) were measured.

**Results:**

The tumor burden experiments in rats bearing estrogen receptor (ER) positive mammary tumours showed that liarozole has a marked anti-tumour effect. In combination with tamoxifen, liarozole had neither an additive nor an antagonistic effect. However, liarozole markedly reduced the uterotrophic effects induced by tamoxifen.

**Conclusion:**

Liarozole's antitumor effects on ER positive mammary tumors and its protective effect on the uterus merit further studies to confirm its clinical value in combination with tamoxifen in ER positive postmenopausal breast cancer. Liarozole and other retinomimetics might also be suitable chemoprevention drugs in combination with tamoxifen because of their favorable toxicity profile.

## Background

Liarozole fumarate (R85246) is an imidazole derivative that inhibits several cytochrome P-450 enzymes, including 4-hydroxylase (which metabolizes retinoic acid) and aromatase, which converts androgens to estrogens in the human placenta, the ovaries and peripheral tissues [1-3]. Liarozole fumarate acts via at least two mechanisms of action: 1) as a powerful third generation aromatase inhibitor and 2) as a retinoic acid metabolism blocking agent (RAMBA) [4-7].

Retinoids (compounds including vitamin A and its synthetic and naturally occurring analogs) are key molecules in cellular proliferation and differentiation [8]. They are known to have a wide variety of effects on cellular differentiation and homeostasis including both anti-carcinogenic and anti-tumoral effects [9-16]. However, prolonged treatment with retinoids is hampered by the induction of retinoid acid (RA) metabolism resulting in a rapid fall in intracellular retinoid levels possibly accounting for their lack of clinical efficacy. This has led to the synthesis of a new class of agents called retinoic acid metabolism blocking agents (RAMBAs), which inhibit the P450-mediated catabolism of RA, leading to sustained increases in intracellular tissue and plasma retinoid levels [17-23].

Tamoxifen is widely used as a single agent for the treatment of both pre- and postmenopausal estrogen receptor (ER) positive breast cancer [24]. The concept of combining a retinoid with an antiestrogen is attractive because active and tolerable agents from both classes are available and they have different and potentially complementary mechanisms of action.

Liarozole induces clinical disease response in advanced breast cancer patients with both heavily pre-treated ER positive and ER negative disease [25,26]. In view of its two independent mechanisms of action as a RAMBA and an AI, and because the toxicities of liarozole and tamoxifen appear to be non-overlapping, there is a rationale for investigating the two drugs in combination. We describe here the effects of liarozole fumarate in combination with tamoxifen on the N-methyl-N-nitrosourea (MNU)-induced rat mammary tumour model and in the ovariectomized rat.

## Methods

### Chemicals

The crystalline powder of liarozole (Janssen Research Foundation, Beerse, Belgium) was dissolved in hydroxypropyl-β-cyclodextrin (Janssen Research Foundation, Beerse, Belgium). Tamoxifen (Sigma Chemical Co., St. Louis, MO, USA) was formulated in purified olive oil (Sigma Chemical Co., St. Louis, MO., USA) by first dissolving it in a small volume of ethanol and then evaporating the ethanol under a stream of purified nitrogen to solubilize the tamoxifen in the olive oil. 17β-estradiol (Sigma Chemical Co., St. Louis, MO, USA) was dissolved in 5% ethanol and 95% saline.

### Tumor burden experiments

Animal care and use conformed with the Guide to the Care and Use of Experimental Animals (Canadian Council on Animal Care), and the animal protocol was approved by the University of Toronto Animal Care Committee. Forty-two-day-old pathogen-free female Sprague-Dawley rats (Indianapolis, IN, USA) were housed at 24°C ± 2°C at 50% humidity with a 12 h light/dark cycle. They were acclimatized for 7 days before the start of the experiment with food and water provided *ad libitum*. At 50 days of age the animals received an intraperitoneal injection of 50 mg/kg N-methyl-N-nitrosourea (MNU) (Ash Stevens, Detroit, MI, USA) dissolved in 0.9% NaCl solution acidified to pH 4 with acetic acid within 20 min of preparation [27,28]. The mammary glands were palpated at weekly intervals starting 4 weeks after MNU administration. Animals bearing one or more tumors greater than 10 mm in diameter were subsequently randomized to one of 6 treatment groups (15 rats per group), and treated for 56 consecutive days. Liarozole and vehicle hydroxypropyl-β-cyclodextrin were given twice daily by oral gavages. Tamoxifen and vehicle olive oil were given once daily by subcutaneous injection. Group 1: controls; Group 2: liarozole 20 mg/kg (L20); Group 3: liarozole 20 mg/kg + tamoxifen 100 μg/kg (L20+T100); Group 4: liarozole 80 mg/kg (L80); Group 5: liarozole 80 mg/kg + tamoxifen 100 μg/kg (L80+T100); Group 6: tamoxifen 100 μg/kg (T100). The location of each tumor, the date of its detection and its size, measured using calipers, were recorded weekly. Tumor burden was calculated using the formula: π × L/2 × *w*/2, mm^2 ^and the total cumulative tumor area was calculated for each animal. After 8 weeks of treatment, rats were euthanized with gaseous carbon dioxide.

### Retinoic acid analysis

At the time the animals were sacrificed, blood was taken for plasma retinoic acid (RA) analysis. RA was extracted from plasma and quantified by UV absorbance after HPLC separation as previously described [29].

### Uterotrophic assay and proliferation experiments

Animal care and use conformed with the Guide to the Care and Use of Experimental Animals (Canadian Council on Animal Care), and the animal protocol was approved by the University of Toronto Animal Care Committee.

Immature (21-day-old, 38–45 g) female Sprague Dawley rats (Indianapolis, IN, USA) were ovariectomized (OVX) 7 days before the start of the experiment. The animals were housed with their mothers as described above. The immature animals were acclimatized for 2 days before being dosed. At 21 days of age the animals were weighed and randomized to 7 treatment groups (7 rats per group) and administered one of the following for 4 consecutive days. Liarozole and vehicle hydroxypropyl-β-cyclodextrin were given twice daily by oral gavage. Tamoxifen, 17β-estradiol and vehicle olive oil were given once daily by subcutaneous injection. Group 1: OVX controls; Group 2: OVX + 17β-estradiol 10 μg; Group 3: OVX + liarozole 20 mg/kg; Group 4: OVX + liarozole 80 mg/kg; Group 5: OVX + tamoxifen 1 mg/kg; Group 6: OVX + liarozole 20 mg/kg +tamoxifen 1 mg/kg; Group 7: OVX + liarozole 80 mg/kg + tamoxifen 1 mg/kg.

### Measurement of uterine weight

On day 5 of treatment rats were sacrificed. The uteri were excised, trimmed free of fat, pierced and blotted to remove excess fluid. The body of the uterus was cut just above its junction with the cervix and at the junction of the uterine horns with the ovaries. The uterus was then weighed (wet weight) [30].

### Uterine histology

10% phosphate buffered formalin-fixed uteri were processed for conventional paraffin embedding. Cross sections (4 μm in thickness) were prepared from both horns of each uterus and stained with hematoxylin and eosin.

### Measurement of epithelial lining cell height

Epithelial lining cell height is expressed as a relative measurement based on calibrations in an ocular micrometer on the microscope. The epithelial height was measured at a magnification of ×125, using a Quantimet 500 MC automated image analysis system (Leica Canada). The image analysis system is attached to an Orthoplan microscope equipped with a ×25 objective and a JVC color camera.

### Proliferation cell nuclear antigen (PCNA)

Immunohistochemical staining of PCNA was performed using the DAKO LSAB+ kit with peroxidase (DAKO Diagnostics Canada Inc. Mississauga, Ontario, Canada) according to the manufacturer's instructions. Briefly, the sections were dewaxed in xylene, rehydrated through graded ethyl alcohol, and treated with retrieval solution (0.01 M citrate-buffer, pH 6.0) at 95°C for 30 min in a boiling water-bath. The sections were then cooled to room temperature for 20 minutes and all subsequent steps carried out at room temperature. Sections were treated with 3% hydrogen peroxide to block endogenous peroxidase activity. The sections were then incubated with monoclonal antibodies DAKO-PCNA, PC10 (diluted 1: 100) (DAKO Diagnostics Canada Inc. Mississauga, Ontario, Canada) for 60 min. After washing with phosphate-buffered saline (PBS) the streptavidin complex was applied, then visualized with diaminobenzidine chromogen solution and counterstained with hematoxylin. The percentage of PCNA nuclei was determined by counting a total number of 300 luminal epithelial cells and 600 stromal and myometrial cells per section. The PCNA index (PI) is the number of positively staining cells per 100 cells evaluated. The cells were counted at a magnification of ×400. Quantitative analysis of immunohistochemical staining of PCNA was performed using the Quantimet 500 MC automated image analysis system (Leica Canada). The image analysis system was attached to an Orthoplan microscope and a JVC color camera.

### Statistical analysis

Data are expressed as the mean ± standard error of the mean (SEM). In the tumor burden experiments, a two-stage linear modeling approach for the analysis of repeated measures was used for the primary analysis (31). An auto-regressive (order-1) covariance structure was used to model the square root transformation of the observed data (31). Uterotrophic assay and proliferation experiments as well as retinoic acid measurements were analyzed using a one-way ANOVA with statistical software (Analyse-it Software, Ltd., UK). Pair-wise comparisons between groups were performed using Fisher's PLSD post-hoc test. P-values of less than 0.05 were considered statistically significant.

## Results and discussion

### Antitumoral effects of liarozole alone or in combination with tamoxifen

**Figure 1:**
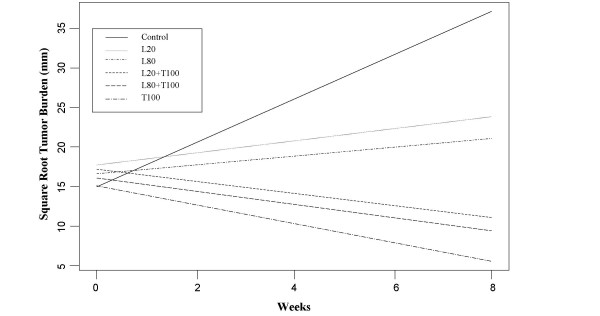
**Effects of liarozole fumarate (R85246) in combination with tamoxifen on N-methyl-N-nitrosourea (MNU)-induced mammary carcinoma and uterus in the rat model**. Effects of liarozole alone and in combination with tamoxifen on tumor burden of MNU-induced rat mammary carcinoma in cycling female Sprague-Dawley rats. Treatment groups: controls, liarozole 20 mg/kg (L20), liarozole 80 mg/kg (L80), liarozole 20 mg/kg in combination with tamoxifen 100 μg/kg (L20+T100), liarozole 80 mg/kg in combination with tamoxifen 100 μg/kg (L80+T100) and tamoxifen 100 μg/kg (T100).

The mean tumor burden for each treatment group is shown in Figure 1. At the end of the treatment period, the average tumor burden area of the L20 (525 mm^2^), L80 (452 mm^2^), L20+T100 (117 mm^2^), L80+T100 (84 mm^2^) and T100 (32 mm^2^) groups, was significantly smaller than in the control animals (1375 mm^2^) (all p < 0.05). The cumulative tumor growth was significantly slower in all treatment groups compared to the control group (p = 0.0001). Both liarozole and tamoxifen when given alone demonstrated anti-tumor effects. However, while tamoxifen lead to tumor shrinkage, liarozole was only able to stop tumor growth. When given in combination, liarozole (80 mg/kg) plus tamoxifen (100 mg/kg) was more effective than liarozole alone (p = 0.0001), Liarozole added to tamoxifen did not alter its anti-tumor effects significantly (p = 0.23). There were no significant differences between the two doses of liarozole either when given alone (p = 0.49) or in combination with tamoxifen (p = 0.8). RA levels varied from 0.09–0.39 ng/ml but were not significantly different between any groups.

Combining either letrozole or anastrozole with tamoxifen lead to a reduction in tamoxifen's anti-tumor effects in preclinical studies [32]. In addition, the combination of anastrozole and tamoxifen was less effective than either agent alone in the large adjuvant ATAC (Anastrozole, Tamoxifen And Combined) study [33]. In view of these data the finding that liarozole did not decrease the anti-tumor effect of tamoxifen in our animal tumor study is of particular importance.

### Uterotrophy and uterine cell proliferation

**Figure 2:**
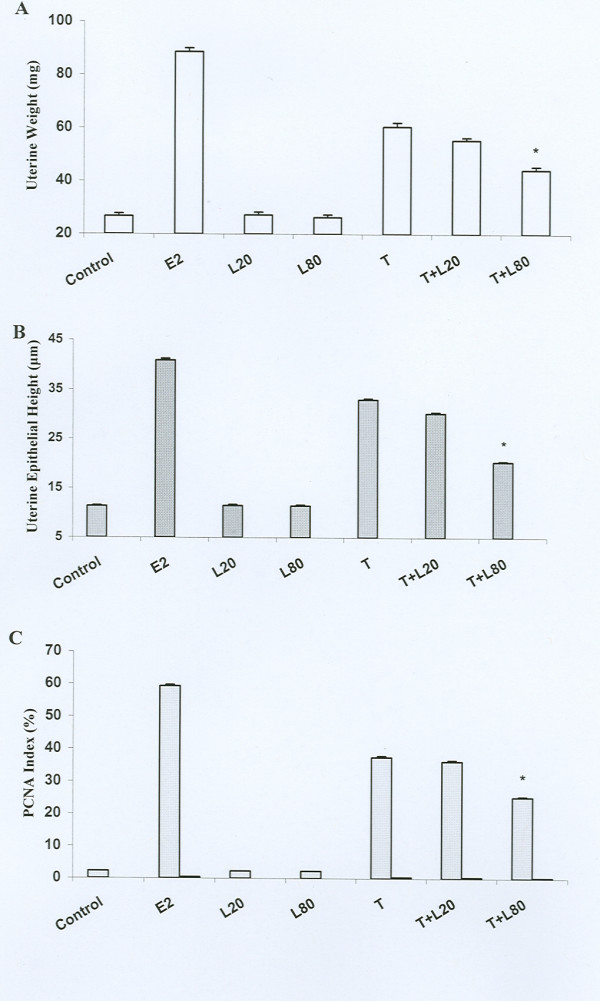
**Effects of liarozole fumarate (R85246) in combination with tamoxifen on N-methyl-N-nitrosourea (MNU)-induced mammary carcinoma and uterus in the rat model**. Effects of liarozole alone and in combination with tamoxifen on uterine wet weight (a), uterine epithelial lining cell height (b) and PCNA index (c). Scale bars represent group mean ± SEM. Treatment groups: OVX controls, 17β-estradiol 10 μg/kg (E_2_), liarozole 20 mg/kg (L20), liarozole 80 mg/kg (L80), tamoxifen 1 mg/kg (T), tamoxifen 1 mg/kg + liarozole 20 mg/kg (T+L20), tamoxifen 1 mg/kg + liarozole 80 mg/kg (T+L80). *P < 0.05 versus tamoxifen 1 mg/kg (T).

**Figure 3:**
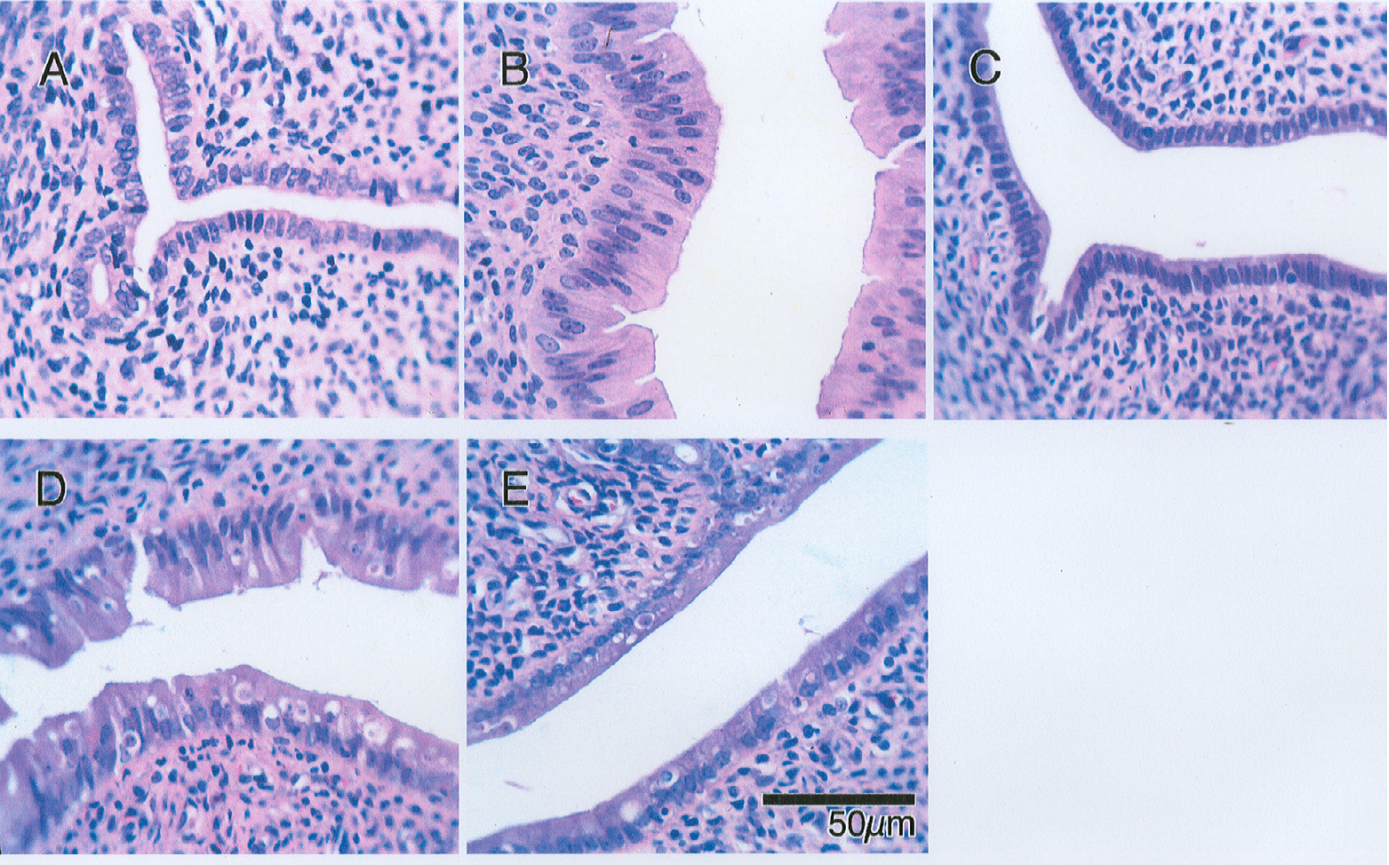
**Effects of liarozole fumarate (R85246) in combination with tamoxifen on N-methyl-N-nitrosourea (MNU)-induced mammary carcinoma and uterus in the rat model**. Hematoxylin and eosin-stained sections of rat uteri illustrating epithelial lining cells obtained from the following groups: OVX controls (A), 17β-estradiol 10 μg/kg (B), liarozole 80 mg/kg (C), tamoxifen 1 mg/kg (D) and liarozole 80 mg/kg + tamoxifen 1 mg/kg (E). Note the inhibitory effect of liarozole significantly negated the tamoxifen stimulatory effect on the epithelial cells (bar in E = 50 μm).

**Figure 4:**
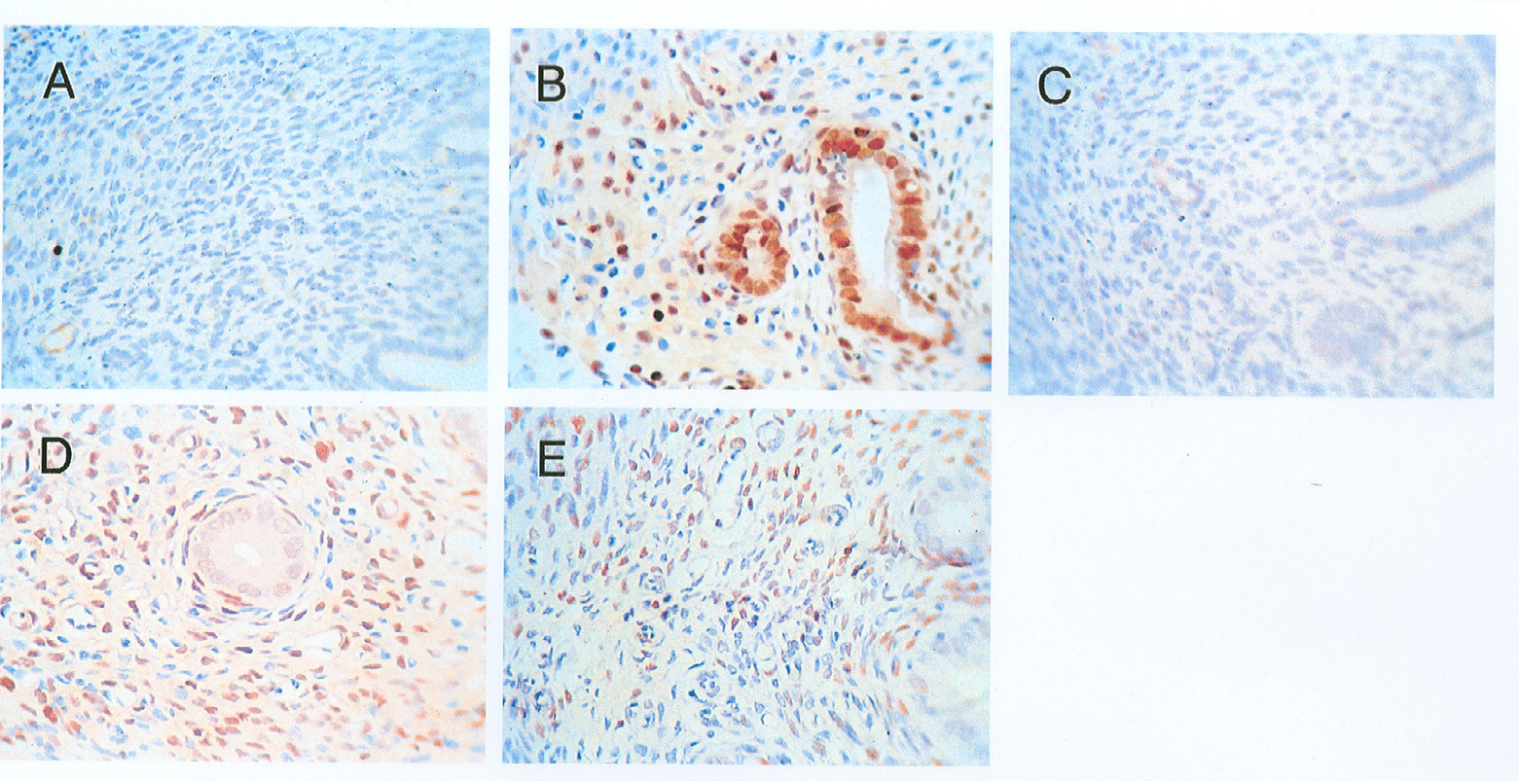
**Effects of liarozole fumarate (R85246) in combination with tamoxifen on N-methyl-N-nitrosourea (MNU)-induced mammary carcinoma and uterus in the rat model**. Immunohistochemical staining for PCNA in the uterus of rats obtained from OVX controls (A), 17β-estradiol 10 μg/kg (B), liarozole 80 mg/kg (C), tamoxifen 1 mg/kg (D) and liarozole 80 mg/kg + tamoxifen 1 mg/kg (E). Note the inhibitory effect of liarozole significantly negated the tamoxifen stimulatory effect on the PCNA staining (magnification, ×400).

The effects of liarozole and tamoxifen alone or in combination on uterine wet weight, epithelial height and PCNA index were all closely correlated with each other (Figure 2). Figures 3 and 4 show photomicrographs, which demonstrate the effects of the different drugs on epithelial height and PCNA, respectively. Both estrogen and tamoxifen alone caused significant increases in uterine wet weight, uterine epithelial height and PCNA index. Both doses of liarozole alone had no significant effect on uterine height, weight or PCNA. When the higher dose of liarozole (80 mg/kg) was combined with tamoxifen it was able to significantly reduce tamoxifen's uterotrophic effects (*P *< 0.05).

These results are consistent with those of Brodie et al., who found that while both letrozole and anastrozole were able to block the effects of estrogen on the uterus, when combined with tamoxifen they reduced its stimulatory effects [32]. It seems that the anti-estrogenic effects of aromatase inhibitors are able to completely antagonize the pro-estrogenic effects of tamoxifen on the endometrium. Although these data show that the combination of tamoxifen and an aromatase inhibitor has an improved toxicity profile compared to tamoxifen, combinations of a pure aromatase inhibitor clinically with tamoxifen are no longer being tested because of the data from the ATAC trial. In contrast to the aromatase inhibitors, our results demonstrate that retinoids do not reduce the SERMs' antitumor effects, offering the possibility to combine these two classes of agents in the clinical setting.

## Conclusion

Liarozole when given alone was able to slow the growth of tumors in the MNU-induced ER positive rat mammary tumor model but we were not able to demonstrate an additive anti-tumor effect of liarozole and tamoxifen. However, in contrast to other third generation aromatase inhibitors, we have shown that liarozole does not reduce tamoxifen's anti-tumor effects. In addition, liarozole is known to have additional inhibitory effects on ER negative tumors [26] and it may reverse tamoxifen resistance [34]. We have also demonstrated that liarozole is able to reduce the unwanted uterotrophic effects of tamoxifen. For these reasons, the clinical combination of liarozole and tamoxifen might be worth evaluating. In particular in the chemopreventive setting, where two of the main goals are to limit toxicities and to target both ER positive and ER negative disease, the combination of these two drugs or novel SERMS in combination with other retinomimmetics merit further study.

## Competing interests

The author(s) declare that they have no competing interests.

## Authors' contributions

PEG designed the study and drafted the manuscript. KS participated in the design of the study and helped to draft the manuscript. SQ conducted the experiments in laboratory, carried out data collection, and helped to draft the manuscript. HH performed the experiments in laboratory. All authors reviewed and approved the final version of the manuscript.

## Pre-publication history

The pre-publication history for this paper can be accessed here:
